# High Potency VEGFRs/MET/FMS Triple Blockade by TAS-115 Concomitantly Suppresses Tumor Progression and Bone Destruction in Tumor-Induced Bone Disease Model with Lung Carcinoma Cells

**DOI:** 10.1371/journal.pone.0164830

**Published:** 2016-10-13

**Authors:** Hidenori Fujita, Akira Gomori, Yayoi Fujioka, Yuki Kataoka, Kenji Tanaka, Akihiro Hashimoto, Takamasa Suzuki, Kenjiro Ito, Tomonori Haruma, Hiromi Yamamoto-Yokoi, Naomoto Harada, Motomu Sakuragi, Nobuyuki Oda, Kenichi Matsuo, Masaki Inada, Kazuhiko Yonekura

**Affiliations:** 1 Discovery and Preclinical Research Division, Taiho Pharmaceutical Co., Ltd., Tsukuba, Ibaraki, Japan; 2 Department of Biotechnology and Life Science, Tokyo University of Agriculture and Technology, Koganei, Tokyo, Japan; Charles P. Darby Children's Research Institute, 173 Ashley Avenue, Charleston, SC 29425, UNITED STATES

## Abstract

Approximately 25–40% of patients with lung cancer show bone metastasis. Bone modifying agents reduce skeletal-related events (SREs), but they do not significantly improve overall survival. Therefore, novel therapeutic approaches are urgently required. In this study, we investigated the anti-tumor effect of TAS-115, a VEGFRs and HGF receptor (MET)-targeted kinase inhibitor, in a tumor-induced bone disease model. A549-Luc-BM1 cells, an osteo-tropic clone of luciferase-transfected A549 human lung adenocarcinoma cells (A549-Luc), produced aggressive bone destruction associated with tumor progression after intra-tibial (IT) implantation into mice. TAS-115 significantly reduced IT tumor growth and bone destruction. Histopathological analysis showed a decrease in tumor vessels after TAS-115 treatment, which might be mediated through VEGFRs inhibition. Furthermore, the number of osteoclasts surrounding the tumor was decreased after TAS-115 treatment. *In vitro* studies demonstrated that TAS-115 inhibited HGF-, VEGF-, and macrophage-colony stimulating factor (M-CSF)-induced signaling pathways in osteoclasts. Moreover, TAS-115 inhibited Feline McDonough Sarcoma oncogene (FMS) kinase, as well as M-CSF and receptor activator of NF-κB ligand (RANKL)-induced osteoclast differentiation. Thus, VEGFRs/MET/FMS-triple inhibition in osteoclasts might contribute to the potent efficacy of TAS-115. The fact that concomitant dosing of sunitinib (VEGFRs/FMS inhibition) with crizotinib (MET inhibition) exerted comparable inhibitory efficacy for bone destruction to TAS-115 also supports this notion. In conclusion, TAS-115 inhibited tumor growth via VEGFR-kinase blockade, and also suppressed bone destruction possibly through VEGFRs/MET/FMS-kinase inhibition, which resulted in potent efficacy of TAS-115 in an A549-Luc-BM1 bone disease model. Thus, TAS-115 shows promise as a novel therapy for lung cancer patients with bone metastasis.

## Introduction

Bone metastasis frequently occurs in patients with cancer, and impairs quality of life and survival. Notably, bone metastasis is reported to occur in 25 to 40% of patients with lung cancer, and indicates poorer prognosis than that in patients with other cancers [1, 2]. Advanced bone metastasis increases the risk of skeletal-related events (SREs), which are defined as the presence of pathological fracture, radiation to the bone, spinal cord compression, or surgery to the bone [3]. Bone modifying agents such as bisphosphonates and denosumab, a fully human antibody that targets RANKL, have improved the occurrence of SREs [4, 5]. However, the contribution of these agents to improvement of overall survival is inadequate. Therefore, novel therapies associated with anti-tumor effects against bone metastasis are urgently required.

HGF-MET and VEGF-VEGFR signaling pathways play important roles in bone metabolism. MET and VEGFR and their ligands, HGF and VEGF, respectively, are expressed in both osteoblasts and osteoclasts [6]. HGF-MET and VEGF-VEGFR signaling has been reported to engage in bone remodeling by promoting osteoclast differentiation/function and upregulating RANKL in osteoblasts [7, 8]. MET and VEGFR signaling also has pivotal roles in cancer progression and bone metastasis. Higher expression of MET was reported in bone metastasis patients [9, 10]. Plasma concentrations of VEGF were increased in patients with positive bone scans or histologic confirmation of cancer metastasis to pelvic lymph nodes [11]. VEGF-VEGFR signaling is well-known to play pivotal roles in tumor angiogenesis [12]. Cabozantinib, a small molecule VEGFRs and MET-targeted kinase inhibitor, has shown improvement in bone pain and reduction in narcotic use in patients with castration-resistant prostate cancer [13, 14]. These insights indicate that simultaneous inhibition of the VEGFR- and MET-axis is a reasonable therapeutic strategy for bone metastasis as targets for both tumor growth and abnormal bone metabolism. In addition to VEGFRs/MET signaling, FMS signaling is reported to have pivotal roles not only in bone metabolism but also in cancer bone metastasis. FMS, which was first discovered as the oncogene responsible for Feline McDonough Sarcoma, is a type III receptor tyrosine kinase that binds to the macrophage or monocyte colony-stimulating factor (M-CSF or CSF-1). Signal transduction as a result of that binding promotes the survival, proliferation, and differentiation of cells of the monocyte/macrophage lineage. Overexpression of CSF-1 and/or FMS has been implicated in a number of disease states such as in the growth and metastasis of certain types of cancer, in the promotion of osteoclast proliferation in bone osteolysis, and in many inflammatory disorders [15].

TAS-115 is a potent VEGFRs and MET-targeted kinase inhibitor, and is currently in a phase I study. We previously reported that TAS-115 showed potent anti-tumor efficacy with higher tolerability compared to pre-existing VEGFRs inhibitors [16]. Herein we identified that TAS-115 is a potent inhibitor of FMS kinase as well as of VEGFRs/MET kinases, and showed its potent anti-tumor efficacy in a tumor-induced bone disease model.

## Materials and Methods

### Cell lines and reagents

A549 cells were purchased from DS Pharma Biomedical (Osaka, Japan). TAS-115 [4-[2-fluoro-4-[[[(2-phenylacetyl)amino]thioxomethyl]amino]-phenoxy] -7-methoxy-N-methyl-6-quinolinecarboxamide] was prepared by Taiho Pharmaceutical Co., Ltd. (Tokyo, Japan). Crizotinib and zoledronic acid were purchased from Daicel Corporation (Tokyo, Japan) and Sigma Aldrich (St. Louis, MO), respectively. Sunitinib was synthesized in our laboratory according to published procedures [17]. Anti-MET antibody was purchased from Santa Cruz Biotechnology, Inc. (Dallas, TX). Anti-phosphorylated MET, anti-VEGFR, anti-phosphorylated VEGFR2, anti-phosphorylated ERK1/2, anti-ERK1/2, anti-phosphorylated AKT, anti-AKT, anti-FAK, anti-S6, anti-STAT3 and anti-GAPDH antibodies were purchased from Cell Signaling Technology (Danvers, MA). Anti-VEGFR antibody for immunoprecipitation was purchased from R&D Systems, Inc. (Minneapolis, MN). Anti-mouse CD31 antibody was purchased from BD Pharmingen^TM^ (Franklin Lakes, NJ). Recombinant human VEGF (rhVEGF) and HGF (rhHGF) were purchased from R&D Systems, Inc. (Minneapolis, MN). RANKL and M-CSF were purchased from the Oriental Yeast Co., Ltd. (Tokyo, Japan), and Kyowa Hakko Kirin Co., Ltd. (Tokyo, Japan), respectively. The TRACP and ALP assay kit was purchased from TAKARA BIO INC (Shiga, Japan). Rabbit anti-Ki-67 antibody was purchased from Abcam (Cambridge, MA). Tartrate-resistant acid phosphatase (TRAP) staining was conducted by using the commercially available TRAP/ALP staining kit (Wako Pure Chemical Industries, Ltd., Osaka, Japan). The PathScan® RTK Signaling Antibody Array Kit (Fluorescent Readout) was purchased from Cell Signaling Technology.

### Establishment of A549-Luc and A549-Luc-BM1 cells

A Hind III-Xba I fragment (1.7 Kb in length) of the luciferase 2 gene was prepared from pGL4.13 (Promega, WI) and inserted into an EcoRV site of pIRESneo (Clontech, CA). The resulting pIRES-Luc was transfected into A549 cells to establish A549-Luc. Luciferase activity was measured using Bright-GLO^TM^ (Promega) according to the manufacturer’s instructions. Under isoflurane anesthesia (1.5–2.5%), A549-Luc cells (10^6^ cells) were injected into the left cardiac-ventricle (LV) of 6 week-old male BALB/c nude mice (CLEA Japan, Tokyo, Japan). Metastatic tumor cells were prepared from a tibia bone and luciferase activity in the cells was confirmed after their culture with G418 at a concentration of 200 μg/mL. The cells recovered from the tibia after LV-injection were highly metastatic to bone compared to the parental A549-Luc cells, and were designated as A549-Luc-BM1. The profile of short tandem repeats of A549-Luc-BM1 cells was consistent with that of the parental A549 or A549-Luc cells (data not shown).

### Tumor-induced bone disease model

Under isoflurane anesthesia (1.5–2.5%), A549-Luc-BM1 cells (2×10^6^ cells) were injected into the right tibia of 6 week-old male BALB/c nude mice. Tumor growth in the tibiae was monitored by bioluminescence imaging using an IVIS Lumina II Imaging System (PerkinElmer, MA). Prior to imaging, the mice were anesthetized with isoflurane (1.5–2.5%) and subsequently _D_-Luciferin potassium salt (Promega KK., WI) was injected intravenously at a concentration of 150 mg/kg. Images were analyzed using the Living Image 3.1 software (PerkinElmer). Total photon flux (TP) was calculated by summation of the photon flux of ventral and right lateral images. One week post tumor implantation, 8–9 animals were allocated to each experimental group by a stratified randomization method with SAS version 9.2 (SAS Institute Japan, Tokyo, Japan) using TP as an allocation parameter, following which drug treatment was then started. TAS-115 (200 mg/kg), cabozantinib (15 mg/kg), sunitinib (40 mg/kg), crizotinib (100 mg/kg), and concomitant dosing of sunitinib (40 mg/kg) with crizotinib (100 mg/kg) were orally administered once a day for 4 weeks. The doses of each drug were selected based on the maximum tolerated dose (MTD) in in-house studies (data not shown). The MTD was defined as the maximum dose that did not result in any animal death or in body weight loss that was more than 10% of the initial body weight. Zoledronic acid (ZA) was subcutaneously injected twice weekly for 4 weeks at a dose of 0.2 mg/kg, which was a previously reported efficacious dose [18]. The allocation day was set as day 0, and drug administration was initiated from day 1. As an indicator of changes in tumor growth during the dosing period, relative total photon flux (RTP), was calculated according to the following formula: RTP_day n_ = (TP on each measurement day) / (TP_day 0_) × 100. TP_day 0_ is the TP on the allocation day. Anti-tumor efficacy was assessed at the end of the study period (day 28) by calculating the tumor growth inhibition percentage (TGI; %) using the following formula: TGI; % = 100 × (1− ((RTP_day 28_ for the treatment group)–(RTP_day 0_)) / ((RTP_day 28_ for the control group)–(RTP_day 0_))).

Changes in body weight (BW) during the dosing period were determined using body weight change (BWC; %) as an indicator, which was calculated using the following formula: BWC; % = 100 × ((BW on each measurement day)–(BW_day 0_)) / (BW_day 0_). BW_day 0_ is the BW on the allocation day. The schedule for the *in vivo* study is depicted in the Supporting Information (S1 Fig).

All animal procedures were done in compliance with National Institutes of Health guidelines and were approved by the Taiho Institutional Animal Care and Use Committee.

### Bone morphometrical analyses by micro-CT

On day 29, the mice were euthanized by cervical dislocation under isoflurane anesthesia, and subsequently the A549-Luc-BM1-implanted tibia was removed and was fixed in 4% paraformaldehyde solution. Micro-CT imaging was performed by using Rm_CT2 (Rigaku corporation, Tokyo, Japan) with the following parameters: 90 kV, 160 μA, 3 min exposure, 10 mm field of view (20 μm voxel size). After scanning, 3D reconstruction was performed to produce a series of cross-sectional images by using specific software (3D Viewer Version 3.00.01, Rigaku corporation). In addition, bone mineral density (BMD) and 3D morphometrical primary parameters, including total volume (TV) and bone volume (BV), were calculated by using bone microstructure software (TRI/3D-BON-FCS64, Ratoc System Engineering, Tokyo, Japan). Volumetric BMD (vBMD), which has been reported to be a good index of osteoporosis [19], was calculated from primary parameters using the following formula: vBMD = BMD × BV / TV.

### Histological analysis

Histological analysis of bone was performed after dosing for 2 weeks, because bone destruction at 5 weeks after implantation of A549-Luc-BM1 was too severe for histological investigation. TAS-115 (100 or 200 mg/kg), crizotinib (100 mg/kg), cabozantinib (15 mg/kg), sunitinib (40 mg/kg), and concomitant dosing of sunitinib (40 mg/kg) with crizotinib (100 mg/kg) were orally administered once a day to A549-Luc-BM1 cell IT-implanted 6–7 week-old male BALB/c nude mice. On day 15, the mice were euthanized by cervical dislocation under isoflurane anesthesia, and their tibiae were collected. For CD31 staining, frozen sections (3 μm thick) were prepared using Kawamoto’s film method [20]. Purified Rat Anti-Mouse CD31 (1:100, BD Pharmingen^TM^) was detected using Simple Stain^TM^ Mouse MAX PO (Rat) (Nichirei) and 3,3’-diaminobenzidine (Dako, Glostrup, Denmark). For Ki-67 and TRAP staining, formalin-fixed paraffin-embedded sections (2 μm thick) were prepared. Anti-human Ki-67 Rabbit Monoclonal Antibody (1:50, Epitomics) was detected using the EnVisionTM^+^ System (Dako). TRAP staining was performed using the TRAP/ALP Stain Kit (Wako Pure chemical Industries, Ltd.). To quantify each staining, whole slides were scanned under 20× magnification with the Aperio Scan-Scope XT Slide Scanner (Aperio Technologies, Inc., CA). To quantify CD31-positive microvessels as microvessel density (MVD, number/mm^2^), four fields of tumor area per section excluding bone and necrotic tissue were analyzed by using Image-Pro Plus (Media Cybernetics, Inc., Carlsbad, CA). The mean values of the four areas were considered as the MVD of each animal. The Ki-67 index was analyzed in the tumor area that was 3 mm in the axial direction from epiphyseal cartilage. Ki-67-positive cells and total tumor cells were counted by using Image-Pro Plus. The Ki-67 index is expressed as a percentage (the number of Ki-67-positive tumor cells / the total number of the tumor cells × 100). The numbers of TRAP-positive osteoclasts were counted, and are expressed as the TRAP-positive osteoclast labeling index, (the number of TRAP-positive cells / the length of the boundary between tumor and trabecular bone (number/mm)). Cells that were TRAP-positive, had more than one nucleus, and were found on the surface of the bone, were identified as osteoclasts. The boundary length was calculated by Image-Pro Plus. All histopathological analyses were conducted in a blinded manner.

### *In vitro* osteoclast differentiation assay

To prepare macrophage colony-stimulating factor (M-CSF) dependent bone marrow macrophages (MDBMs), bone marrow cells were obtained from the femurs of male BALB/c mice (CLEA Japan) and adherent cells were cultured with human M-CSF (hM-CSF; 10000 U/mL) for 6 days. MDBMs were collected and cultured with hM-CSF (10000 U/mL) and recombinant human soluble RANKL (rhsRANKL)-GST (5 nM) for 4 days (25000 cells/well in a 96 well flat plate) for induction of differentiation into osteoclasts. Test compounds were then added to the culture media at the indicated concentration. TRAP activities were measured using the commercially available TRACP and ALP Assay Kit (TAKARA BIO INC).

### *In vitro* MDBM signaling

For analysis of the expression and phosphorylation levels of FMS, MET and VEGFR2, MDBMs were cultured with hM-CSF (20000 U/mL) for 4 days (400000 cells/dish in Suspension Culture Dishes (Corning Inc., NY). The phosphorylation levels of FMS and downstream signaling molecules were then analyzed in these MDBMs following incubation for 1 hr with test compounds at the indicated concentration. The phosphorylation of MET or VEGFR2 was analyzed in these MDBMs following stimulation with rhHGF (100 ng/mL, 10 min) or rhVEGF (100 ng/mL, 5 min), respectively. After incubation, the MDBMs were harvested, the cells were lysed and target signaling proteins were detected by immunoblotting. For analysis of VEGFR2 and its phosphorylated form, the lysate was immunoprecipiated with an anti-VEGFR antibody prior to immunoblotting.

### *In vitro* kinase assay

Recombinant human FMS dephosphorylated with lambda phosphatase (dephospho-rhFMS, N-terminal His-tagged, 538–972 amino acids) was obtained from Carna Biosciences, Inc. (Hyogo, Japan). Enzyme inhibition studies were performed using LANCE^®^ Ultra TR-FRET assay technology [21]. Briefly, 0.03 μg/mL dephospho-rhFMS and 100 nM ULightTM-poly GT (4:1) (PerkinElmer) were mixed and incubated for 40 min at 25°C in 10 μL of reaction mixture containing 100 μM ATP, 15 mM tris (hydroxymethyl) aminomethane (Tris (pH 7.5)), 0.01% (v/v) Tween 20, 5 mM MgCl_2_, 2 mM dithiothreitol and various concentrations of the test compound. The reaction was terminated by addition of 5 μL of 120 mM ethylenediaminetetraacetic acid (EDTA), followed by addition of 5 μL of a detection mixture containing 8 nM LANCE^®^ Eu-W-1024 labeled anti-phosphotyrosine PT66 antibody (PerkinElmer) in 30 mM Tris (pH 7.4) and 0.2% Tween 20. After incubation for 60 min at room temperature, phosphorylation of the substrate peptide was monitored by measurement of the TR-FRET signal under excitation at 337 nm with the PHERAstar FS microplate reader (BMG LABTECH GmbH, Ortenberg, Germany). By referring to the TR-FRET signals of positive (no inhibitor) and negative (EDTA was added before the reaction was started) control wells, the percent inhibition of each well was calculated and the half-maximal inhibitory concentration (IC_50_) value was determined using a four-parameter sigmoidal equation. The assay of MET and VEGFR2 kinase activity was performed as described previously [16].

### Phospho-RTK array analysis

A549-Luc-BM1 cells (5 × 10^5^ cells/dish) were plated with conditioned medium. On the following day, the medium was replaced with RPMI1640 medium containing 10 mM HEPES and 0.2% (v/v) fetal bovine serum, and the cells were incubated overnight. HGF was then added at a final concentration of 100 ng/mL and the culture plate was incubated for 15 min. After washing with PBS, the cells were lysed using the cell lysis buffer provided. The cell lysate was applied to the array slides. Fluorescent signals from the array slides were detected by the Odyssey Infrared Imaging System (LI-COR).

### Statistical analysis

Statistical analysis was performed using Student’s *t*-test and Dunnett’s multiple comparison test with SAS version 9.2 (SAS Institute Japan, Tokyo, Japan). A p value less than 0.05 was considered to be statistically significant.

## Results

### Establishment of A549-Luc-BM1 cells

**Fig 1:**
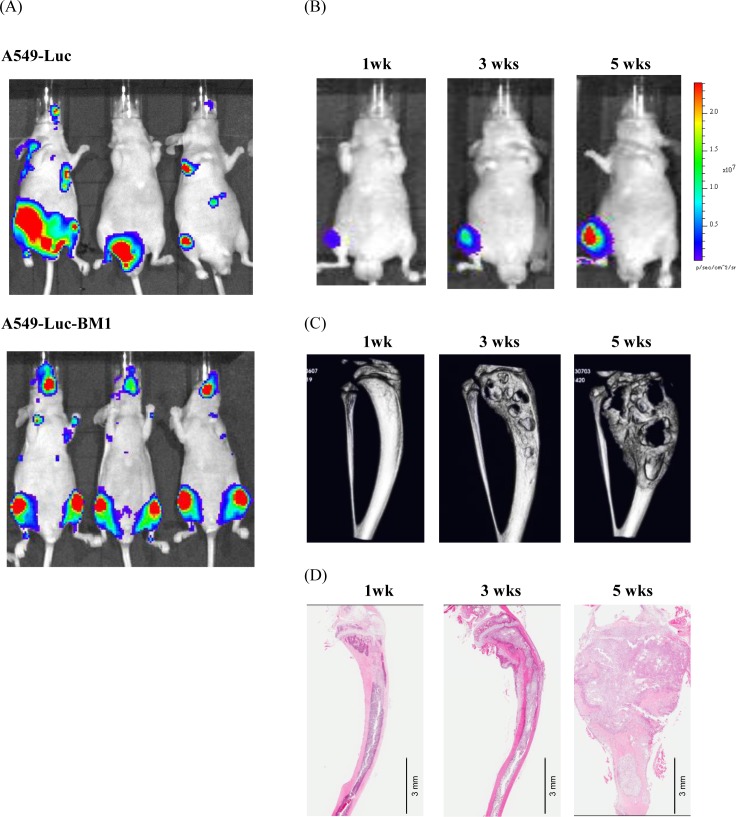
Characterization of the mouse bone disease model with A549-Luc-BM1 cells. (A) Representative pictures of metastatic site difference between parental A549-Luc and A549-Luc-BM1 cells after left cardiac-ventricle implantation into mice. (B) Representative optical changes after intra-tibial implantation of A549-Luc-BM1 cells. (C) Micro-CT images of A549-Luc-BM1 implanted mouse tibia. (D) Hematoxylin and Eosin staining of A549-Luc-BM1 implanted mouse tibia. Each picture was taken from different mice that were selected based on average data at each time point. A549-Luc-BM1 cells caused tumor growth in bone and aberrant bone remodeling after their implantation. Hematoxylin and Eosin staining demonstrated that the tumor protruded from the tibia at 5 weeks after implantation. Scale bar indicates 3 mm.

To establish a bone disease model, A549 human lung adenocarcinoma cells were transfected with the luciferase gene and injected into the LV of BALB/c nude mice. Although bioluminescence signals from tumor cells were detected in bone tissue such as in the femur, pelvis, spine and mandible, tumor cells also metastasized to other organs (Fig 1A). To remove mouse interstitial cells, A549-Luc cells were collected from the metastasized tibia and cultured *in vitro* with G418. When the recovered cells were re-injected into the LV, they were predominantly and efficiently metastasized to bone compared to the parental A549-Luc cells (Fig 1A), and were designated as A549-Luc-BM1 cells. The 50% growth inhibitory concentration (GI_50_) of TAS-115 for A549-Luc-BM1 cells was 11.2 μM (S2 Fig). Although the MET phosphorylation levels in A549-Luc-BM1 cells were low under normal culture conditions, exogenous HGF induced the phosphorylation of MET (S3 Fig). The bioluminescence signal from A549-Luc-BM1 cells that was detected using an *in vivo* imaging system was correlated with cell number *in vitro* (data not shown).

### IT mouse bone disease model using A549-Luc-BM1 cells

When A549-Luc-BM1 cells were injected into the LV, the cells were predominantly metastasized to bone. However, the target sites of bone metastases were multiple and random. It was possible that this profile of A549-Luc-BM1 cells might cause considerable variation in tumor growth at each metastasized site, which would make it difficult to conduct detailed analysis of the efficacy of agents on bone and their mode of action. To avoid this problem, A549-Luc-BM1 cells were locally implanted into the proximal tibia of nude mice and the tumor growth was monitored by using an *in vivo* imaging technique. Tumor burden within the tibia progressed so rapidly that the bioluminescence signal at 5 weeks was 86-fold higher than that at 1 week post tumor implantation (Fig 1B). Tumor progression was accompanied by severe osteolytic/osteoblastic lesions as well as by cortical destruction that were observed by micro-CT analysis (Fig 1C). Hematoxylin-eosin staining showed that the tumor filled the marrow space inside the tibia until 3 weeks post tumor implantation, then destroyed the bone structure and finally protruded into the surrounding muscle at 5 weeks post tumor implantation (Fig 1D).

### TAS-115 suppressed the growth of A549-Luc-BM1 cells in the mouse tibia

**Fig 2:**
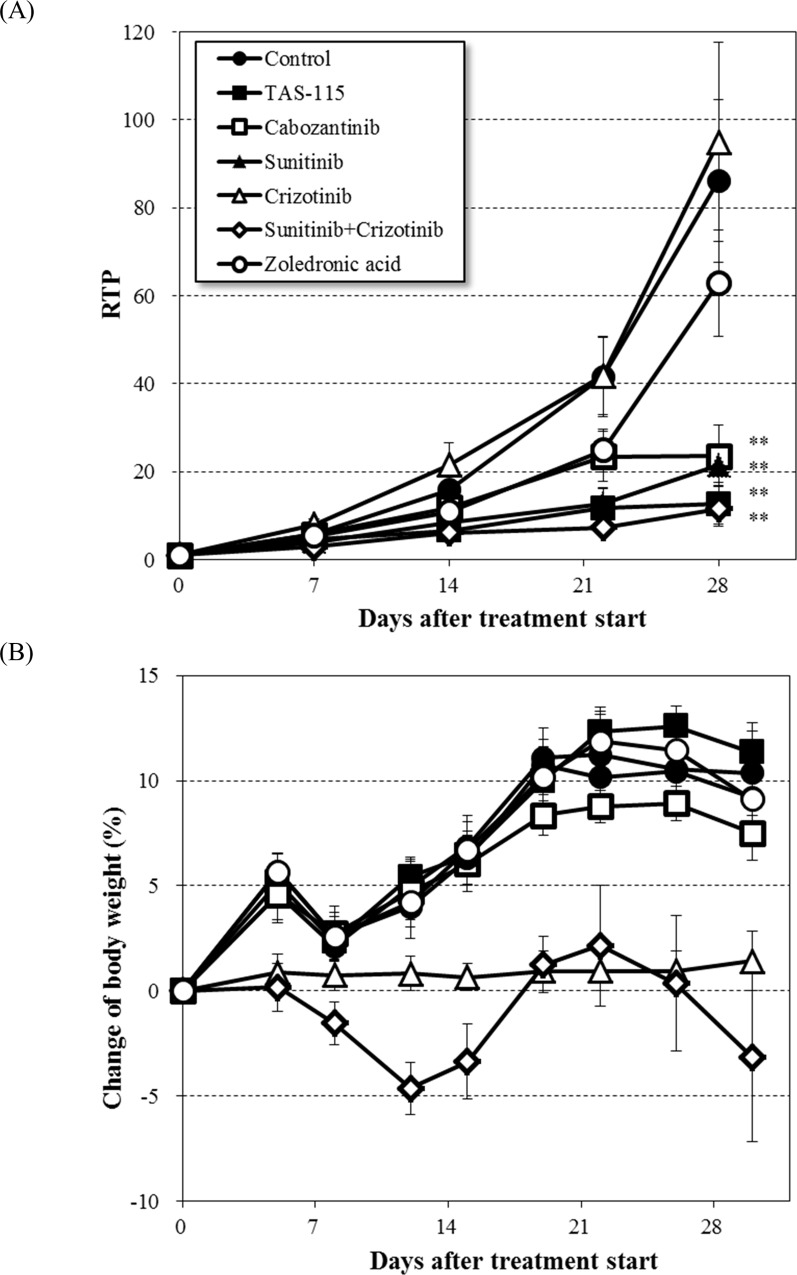
Anti-tumor efficacy of TAS-115 in the bone disease model of A549-Luc-BM1 in mice. (A) The changes in bioluminescence signals after oral administration of each of TAS-115 (200 mg/kg), cabozantinib (15 mg/kg), sunitinib (40 mg/kg), crizotinib (100 mg/kg), or the combination of sunitinib and crizotinib, or subcutaneous administration of zoledronic acid (0.2 mg/kg) for 4 weeks following intra-tibial implantation of A549-Luc-BM1 cells in mice. Relative total photon flux (RTP) was calculated according to the following formula: RTPn = (TP on each measurement day) / (TP _day 0_) ×100. Data are expressed as means ± SE (n = 8–9). **, p<0.01 in the comparison of the treated group with the control group (Dunnett’s test). (B) Body weight changes over the 4 weeks of treatment. Data are expressed as means ± SE (n = 8–9).

The anti-tumor efficacy of TAS-115 on a metastasized tumor in bone was evaluated by using *in vivo* bioluminescence imaging following IT transplantation in the A549-Luc-BM1 model. TAS-115, cabozantinib, and sunitinib each significantly suppressed tumor growth of A549-Luc-BM1 cells in the mouse tibia (TAS-115; TGI = 85%, p<0.01, cabozantinib; TGI = 73%, p<0.01, sunitinib; TGI = 75%, p<0.01, Fig 2A). Crizotinib, a MET inhibitor, did not impact on tumor growth at all in this model, but did suppress body weight gain (Fig 2A). ZA, used as a therapeutic agent against the bone lesion, tended to suppress tumor growth (TGI = 27%). Concomitant dosing of sunitinib with crizotinib markedly inhibited tumor growth (TGI = 86%, p<0.01), which was comparable to the effect of TAS-115 or sunitinib treatment. However, the combination treatment affected body weight gain in mice (Fig 2B).

### TAS-115 attenuated tumor-induced bone destruction in the mouse bone disease model using A549-Luc-BM1

**Fig 3:**
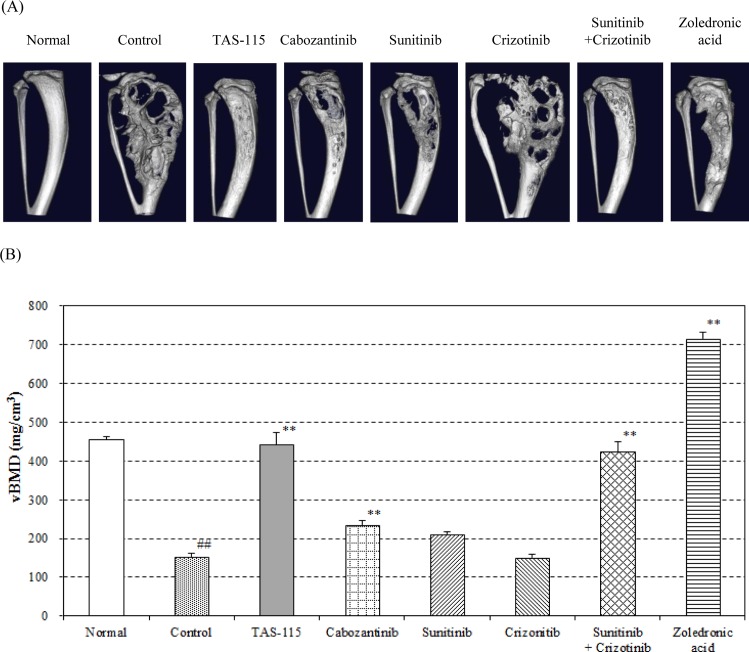
Micro-CT imaging and bone morphometrical analyses after TAS-115 treatment in the A549-Luc-BM1 bone disease model. The A549-Luc-BM1 implanted tibia was removed from the mice after oral administration of TAS-115 (200 mg/kg), cabozantinib (15 mg/kg), sunitinib (40 mg/kg), crizotinib (100 mg/kg), or the combination of sunitinib and crizotinib, or subcutaneous administration of zoledronic acid (0.2 mg/kg) for 4 weeks, and was analyzed using micro-CT. (A) Representative micro-CT image of each treatment group. (B) Volumetric bone mineral density (vBMD) of each group that was calculated using the following formula: vBMD = BMD (bone mineral density) × BV (bone volume) / TV (total volume). Data are expressed as means ± SE (n = 8–9). ##, p<0.01 in the comparison of the treated group with the normal group (Student’s *t*-test). **, p<0.01 in the comparison of the treated group with the control group (Dunnett’s test).

To evaluate the efficacy of TAS-115 on A549-Luc-BM1-related bone destruction in detail, we performed a detailed analysis of IT implantation in the A549-Luc-BM1 model using micro-CT. At five weeks post tumor implantation in bone, A549-Luc-BM1 cells created mixed osteolytic/osteoblastic lesions in the bone (Fig 3A). These changes were characterized by changes in bone parameters. In the metastasized tibia, both bone volume (BV) and total volume (TV) parameters were increased whereas volumetric bone mineral density (vBMD) was decreased compared to the normal tibia (p<0.01, Fig 3B, S1 Table). Sunitinib significantly inhibited A549-Luc-BM1-induced changes in BV and TV, but not in BMD or vBMD (p<0.01, S1 Table). Crizotinib did not affect any changes in the parameters induced by A549-Luc-BM1. However, concomitant dosing of sunitinib with crizotinib significantly suppressed all of the changes in the parameters induced by A549-Luc-BM1 (p<0.01, Fig 3B, S1 Table). Treatment with TAS-115 or cabozantinib also significantly suppressed all of the changes in these parameters (p<0.01, Fig 3B, S1 Table). Notably, TAS-115 alone potently improved bone parameters, and the effect was comparable to that of the combination treatment of sunitinib and crizotinib. In the case of ZA, TV was significantly decreased compared with the control group (p<0.01, S1 Table). However, BMD, BV, and vBMD were all significantly increased by ZA compared with control (p<0.01, S1 Table), and the value of each parameter was higher than that of the normal group.

### TAS-115 suppressed angiogenesis, tumor cell proliferation, and osteoclast accumulation in bone lesions

**Fig 4:**
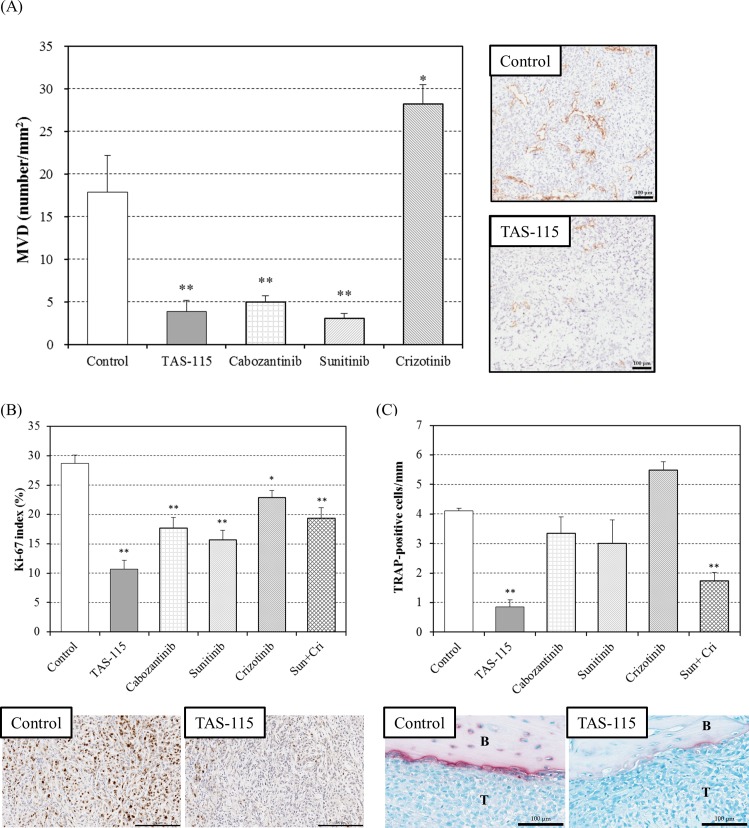
Histological examination after TAS-115 treatment in the bone disease model with A549-Luc-BM1 cells. (A) Tumor microvessel density (MVD) in the mouse bone disease model after oral administration of TAS-115 (100 mg/kg), cabozantinib (15 mg/kg), crizotinib (100 mg/kg), or sunitinib (40 mg/kg) for 2 weeks. The Y axis indicates MVD in A549-Luc-BM1-implanted tibiae. Data are expressed as means ± SE (n = 4–5). * and **, p<0.05 and p<0.01, respectively, in comparisons of the treated group with the control group, (Dunnett’s test). Scale bar indicates 100 μm. (B) Ki-67 staining of A549-Luc-BM1-implanted tibiae. TAS-115 (200 mg/kg), cabozantinib (15 mg/kg), sunitinib (40 mg/kg), crizotinib (100 mg/kg), and the combination of sunitinib (40 mg/kg) and crizotinib (100 mg/kg) were administered orally for 2 weeks. Data are expressed as means ± SE (n = 5). * and **, p<0.05 and p<0.01, respectively, in the comparison of the treated group with the control group (Dunnett’s test). Scale bar indicates 200 μm. (C) TRAP staining of A549-Luc-BM1-implanted tibiae. TAS-115 (200 mg/kg), cabozantinib (15 mg/kg), sunitinib (40 mg/kg), crizotinib (100 mg/kg), and the combination of sunitinib (40 mg/kg) and crizotinib (100 mg/kg) were administered orally for 2 weeks. The red arrows indicate TRAP-positive osteoclasts. Data are expressed as means ± SE (n = 5). B: Bone, T: Tumor (A549-Luc-BM1). **, p<0.01 in the comparison of the treated group with the control group (Dunnett’s test). Scale bar indicates 100 μm.

Histological and immunohistochemical analyses were conducted independently of the anti-tumor efficacy study to investigate the mechanism of action of TAS-115 in the bone disease model of A549-Luc-BM1. TAS-115, cabozantinib, and sunitinib each significantly reduced MVD within the tumor area in the bone lesion (p<0.01, Fig 4A). In contrast, crizotinib did not affect MVD (Fig 4A). To investigate the effect of TAS-115 on tumor proliferation in the tibia, the Ki-67 index was measured. Treatment agents except for crizotinib significantly decreased the Ki-67 index as compared with the control group (p<0.01, Fig 4B). Furthermore, TRAP-positive osteoclasts lined the boundary between the tumor and bone in the control group (Fig 4C), whereas few TRAP-positive cells were observed at the boundary between bone and bone marrow (S4 Fig). TAS-115 significantly decreased the number of osteoclasts in this area (p<0.01, Fig 4C). Interestingly, although single treatment of sunitinib or crizotinib did not decrease the number of osteoclasts, concomitant dosing of sunitinib with crizotinib significantly decreased it (p<0.01, Fig 4C). Cabozantinib did not affect the number of osteoclasts (Fig 4C).

### TAS-115 inhibited osteoclast differentiation and signaling pathways

**Fig 5:**
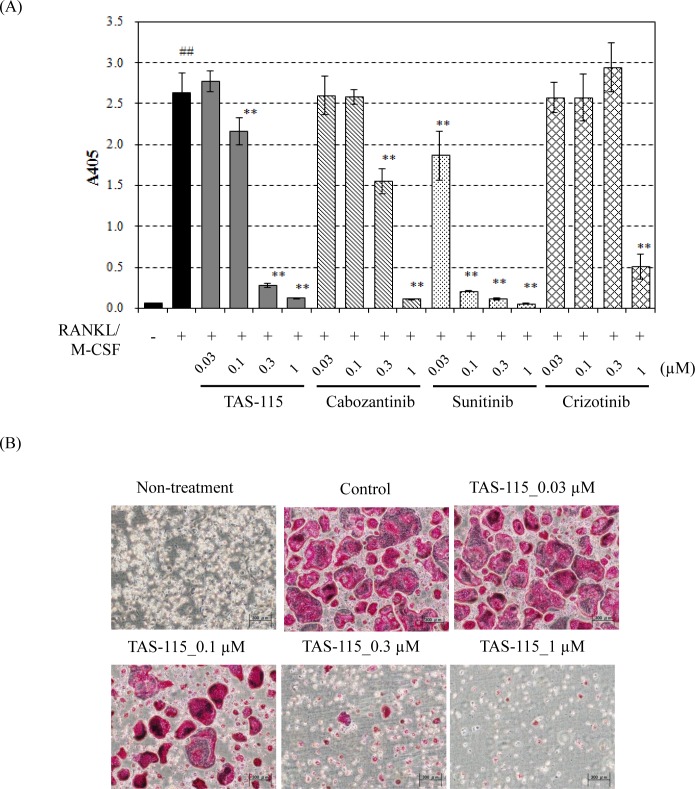
Inhibitory activity of TAS-115 against osteoclast formation and signal transduction in osteoclasts. (A) Effect of TAS-115, cabozantinib, sunitinib, or crizotinib against M-CSF and RANKL induced osteoclast formation. The Y axis indicates absorbance at 405 nm (A405) that reflects TRAP activity in cells, which was used as an indicator of osteoclast formation. Data are expressed as means ± SD (n = 4). ##, p<0.01 in the comparison of the control group (the cultures treated with RANKL and M-CSF) with the non-treated group (the cultures without RANKL and M-CSF treatment) (Student’s *t*-test). **, p<0.01 in the comparison of the treated group with the control group (Dunnett’s test). (B) Representative photographs of TRAP-positive cells in the non-treated, control and TAS-115 treated cultures. M-CSF dependent bone marrow macrophages (MDBMs) were cultured for 4 days without RANKL, M-CSF or TAS-115 as the non-treated group. MDBMs were maintained in M-CSF and RANKL without TAS-115 for 4 days as the control group. For TAS-115 treatment, MDBMs were maintained in M-CSF, RANKL and the indicated concentration of TAS-115 for 4 days.

**Fig 6:**
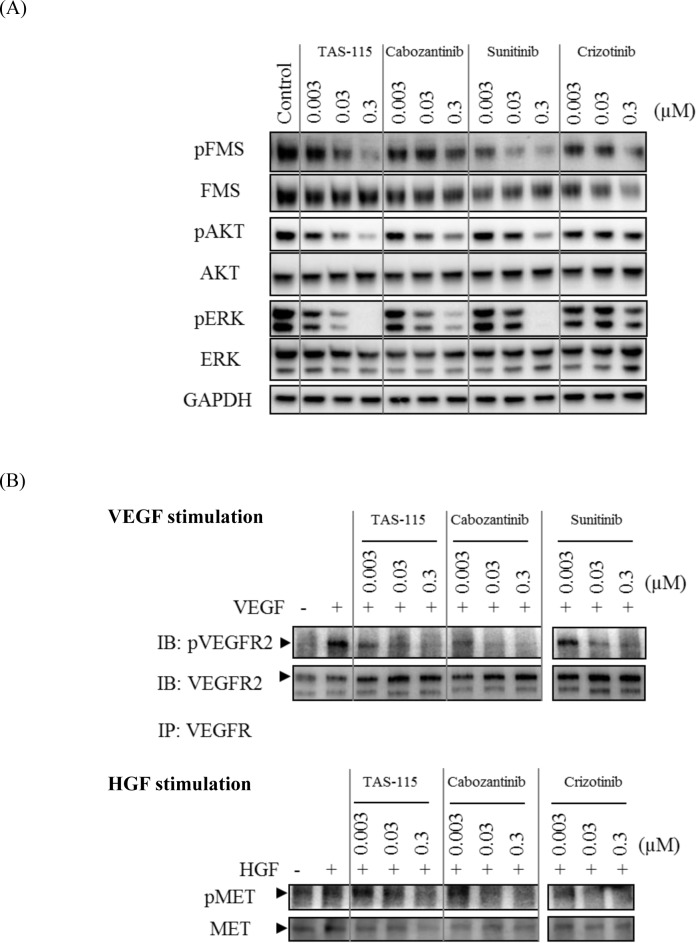
Inhibitory activity of TAS-115 against FMS, VEGFR2 and MET expressed in MDBMs. (A) Effect of TAS-115, cabozantinib, sunitinib, or crizotinib on FMS kinase mediated phosphorylation in MDBMs. MDBMs were cultured for 4 days with M-CSF in plastic dishes. Test compounds were then added at the indicated concentration to the culture media and incubated for 1 hr. Following incubation of MDBMs with hM-CSF and the indicated drugs, cell lysates were then prepared, and analyzed by subsequent Western blotting with the indicated antibodies. (B) Effect of TAS-115 and cabozantinib on ligand induced VEGFR2 and MET phosphorylation in MDBMs. Cell culture and drug treatments were performed as for the experiments with M-CSF. rhHGF (100 ng/mL) or rhVEGF (100 ng/mL) was then added to the culture media, and, following incubation for 10 or 5 min, respectively, cell lysates were prepared. VEGFR2 and its phosphorylated form were detected in MDBMs by Western blotting following immunoprecipitation with an anti-VEGFR antibody. MET and its phosphorylated form were directly detected by Western blotting. Sunitinib and crizotinib were used as positive control agents for VEGFR2 inhibition and MET inhibition, respectively.

Based on the results of the histological analysis, the effect of TAS-115 on osteoclast differentiation was investigated in *in vitro* studies. RANKL and M-CSF are known to induce the differentiation of bone marrow macrophages into osteoclasts [22]. Therefore, the inhibitory potency of TAS-115 towards osteoclast differentiation was evaluated by analysis of TRAP activity in cells after RANKL and M-CSF stimulation. TAS-115 dose-dependently suppressed osteoclast differentiation induced by RANKL and M-CSF, and potently inhibited mouse osteoclast formation at a concentration of 0.3 μM (Fig 5A and 5B). These data revealed that TAS-115 had the potential to affect RANKL or M-CSF-related signaling pathways in mouse osteoclast formation. Sunitinib also potently inhibited RANKL and M-CSF-induced mouse osteoclast formation at a concentration of 0.1 μM. Crizotinib and cabozantinib markedly affected mouse osteoclast formation at a concentration of 1 μM. We additionally investigated the effect of TAS-115 on cellular FMS signaling in MDBMs. Western blotting indicated that M-CSF induced the phosphorylation of FMS and its downstream signaling pathways in MDBMs (Fig 6A). TAS-115 markedly inhibited M-CSF-stimulated phosphorylation of FMS, ERK1/2 and AKT in MDBMs at a concentration greater than 0.03 μM. This inhibition was comparable to that resulting from sunitinib, which was applied as a positive control agent (Fig 6A). In contrast, the inhibition by cabozantinib or crizotinib was moderate even at a concentration of 0.3 μM. To confirm the inhibitory activity of TAS-115 towards human FMS, the cellular IC_50_ value of TAS-115 against M-CSF-induced FMS phosphorylation in human acute monocytic THP-1 leukemia cells was determined (S5 Fig). The IC_50_ value of TAS-115 was 0.012 μM, and no species differences were observed between mouse and human in the inhibitory activity of TAS-115 for FMS. In addition to FMS expression, the expression of MET and VEGFR2 was also detected in MDBMs, and these receptors were activated by exogenous rhHGF and rhVEGF, respectively (Fig 6B). Both TAS-115 and cabozantinib inhibited the phosphorylation of MET and VEGFR2 at a concentration greater than 0.03 μM. Since the inhibitory effect of TAS-115 against VEGFR2 and MET has already been reported [16], we here investigated the inhibitory activity of TAS-115 towards FMS kinase activity using recombinant dephospho-FMS kinase. Sunitinib and TAS-115 potently inhibited FMS activity with IC_50_ values of 0.0035 μM and 0.015 μM, respectively, while cabozantinib did so more weakly with an IC_50_ value of 0.079 μM (Table 1). A549-Luc-BM1 cells did not express human HGF under either *in vitro* or *in vivo* conditions (data not shown). However, we found that both mouse HGF and human VEGF were detectable in A549-Luc-BM1 implanted tibia and that the expression of these growth factors tended to increase with tumor progression in the tibia (S6 Fig). Mouse VEGF was not measured, since human VEGF is known to stimulate the proliferation and survival signal in mouse endothelial. Furthermore, A549-Luc-BM1 cells displayed an approximately 3-fold increase in M-CSF expression compared to A549-Luc cells (p<0.01, S7 Fig). These results suggested that VEGF, HGF, and M-CSF in the bone lesion activated their respective receptor expressed in osteoclasts, and that TAS-115 blocked the lytic activity and survival of osteoclasts through inhibition of VEGFRs/MET/FMS-related signaling pathways.

**Table 1:** Kinase inhibitory activity of TAS-115 for FMS, MET and VEGFR2.

Kinases	IC_50_ (μM)			
	TAS-115	Cabozantinib	Sunitinib	Crizotinib
**FMS (dephospho)**	0.015	0.079	0.0035	0.23
**MET**	0.032	0.032	> 3.0	0.016
**VEGFR2**	0.030	0.012	0.018	> 3.0

## Discussion

In the present study, we established a highly bone-metastatic A549 subline, A549-Luc-BM1 cells, by *in vivo* selection from bone-metastatic lesions resulting from LV injection of A549-Luc cells. When re-injected into the LV, A549-Luc-BM1 cells preferentially metastasized to bone tissue such as femur, pelvis, spine, and mandible with 100% incidence, and produced both osteolytic and osteoblastic lesions. These characteristics are similar to other previously reported highly metastatic sublines [23, 24]. We used this cell line to evaluate the effect of TAS-115 on metastasized tumor growth in bone. After implantation into mouse tibia, A549-Luc-BM1 cells showed tumor growth in bone and created mixed osteolytic/osteoblastic bone destruction. It has been reported that bone metastasis of lung cancer mainly causes osteolytic lesions, but shows mixed lesions and osteoblastic lesions with an incidence of about 15% and 5% of bone metastasis of lung cancer patients, respectively [25]. This property of our model might originate from properties of A549 cells that have been reported in previous studies [26]. Histopathological analysis demonstrated that osteolytic bone destruction was accompanied by an increased number of osteoclasts on the tumor-bone border. Osteoclasts on the tumor-bone border appeared to be induced by A549-Luc-BM1 cells implanted in tibia, because few osteoclasts were observed in the boundary between bone and bone marrow in sham-operated control mice. The phenotypic and histological data suggested that the IT bone disease model using A549-Luc-BM1 cells mimics bone metastasized tumors in a clinical situation, and would be a good preclinical model for the evaluation of anti-tumor drugs.

Herein we demonstrated that TAS-115 strongly suppressed tumor growth and the related bone destruction that occurred by IT implantation of A549-Luc-BM1 cells, while it did not cause body weight loss throughout the study in mice. Histopathological analyses demonstrated that TAS-115 significantly reduced CD31-positive vessels and Ki-67-positive proliferating cells in bone lesions (Fig 4A and 4B). We previously reported that TAS-115 showed potent VEGFRs-kinase inhibition *in vitro*, and reduced CD31-positive vessels in a subcutaneously implanted xenograft model [16]. In contrast, MET signaling might not be involved in tumor growth in this model, since the phosphorylation level of MET was relatively low in A549-Luc-BM1 cells (S3 Fig), and since TAS-115 barely suppressed A549-Luc-BM1 cell proliferation *in vitro* (S2 Fig). Although crizotinib, a MET-targeted inhibitor, induced weak growth inhibition of A549-Luc-BM1 cells (GI_50_ value = 1.1 μM), this effect was considered to be independent of MET inhibition, because crizotinib displayed higher potency against cancer cells with MET amplification (e.g. MKN45, Hs746T, or NUGC4 cells) than against A549-Luc-BM1 cells [16]. Furthermore, crizotinib alone had no effect on tumor growth in the IT bone disease model using A549-Luc-BM1 cells (Fig 2A). These results suggested that TAS-115 suppressed tumor growth in bone via inhibition of VEGF-related tumor angiogenesis rather than via MET inhibition. The fact that sunitinib, a VEGFR inhibitor, also inhibited tumor growth and decreased CD31-positive vessels also supports this notion.

In addition to its effects on the tumor, TAS-115 also impacted on the tumor-related abnormal bone microenvironment. Histopathological analysis revealed that TAS-115 most potently reduced TRAP-positive osteoclast formation around tumor tissue in the tibia (Fig 4C). Although crizotinib alone had no effect on bone destruction in the A549-Luc-BM1 bone disease model, crizotinib enhanced the effects of sunitinib against osteolytic bone destruction (Fig 3) and osteoclast formation (Fig 4C), thus MET signaling is considered to have a supportive role in the development of bone lesions. Moreover, both mouse HGF and human VEGF were detected in A549-Luc-BM1-implanted tibiae, and TAS-115 clearly blocked ligand-induced phosphorylation of the MET- and VEGFR2-kinases in MDBMs *in vitro* (S6 Fig and Fig 6B). HGF has been reported to be secreted by stroma, smooth muscle cells, osteoblasts, and osteoclasts [6, 27–29], functioning as a substitute for M-CSF to support osteoclast differentiation with RANKL, and stimulating osteoclastic resorption in the presence of osteoblasts [30, 31]. Cabozantinib has been reported to inhibit tumor growth in bone in both preclinical and clinical studies, and a direct effect of cabozantinib on osteoblasts is responsible for its anti-tumor efficacy [13]. ARQ-197, a selective MET inhibitor, inhibited bone disease in a mouse LV injection model of 1833/TGL cells without growth inhibition against its subcutaneous xenografts [32]. Furthermore, VEGF-VEGFR signaling was also reported to enhance osteolytic activity and survival of osteoclasts [33]. The expression of VEGFR2 was significantly enhanced in *in vitro* differentiation of osteoclasts from mononuclear precursors [34]. Additionally, VEGF induced osteoclast differentiation and increased the expression of RANK in osteoclast precursor cells [8, 35]. VEGF has been related to the regulation of RANKL expression in osteoblasts [8]. Our results suggested that VEGFRs/MET inhibition might be involved in the reduced number of osteoclasts by TAS-115 in IT A549-Luc-BM1 transplanted model, and the above-referenced reports strongly support a relationship between the VEGFRs- and MET-signaling axis and bone metabolism besides the canonical pathway that is mediated by RANKL and M-CSF signaling. Therefore, targeting of VEGFRs- and MET-signaling pathways plus inhibition of the canonical pathway mediating osteoclast differentiation/function have been reasonable therapeutic strategies for bone metastasis treatment [36].

We identified that TAS-115 had potent inhibitory activity towards FMS kinase as well as VEGFRs and MET kinase (Table 1). M-CSF/FMS signaling plays a crucial role with RANKL in a canonical pathway for osteoclast formation [36]. Overexpression of CSF-1 and/or FMS has been implicated in a number of disease states such as in the growth and metastasis of certain types of cancer, in the promotion of osteoclast proliferation in bone osteolysis, and in many inflammatory disorders [37–39]. Hung et al. showed that M-CSF potentiated lung cancer bone metastasis and that CSF-1R (FMS) knockdown in A549 cells reduced bone metastasis in a preclinical model [40]. Small molecule FMS inhibitors and anti-FMS antibodies have recently been developed for cancer treatment [41, 42]. We showed that A549-Luc-BM1 cells have an approximately 3-fold increase in M-CSF expression compared to the parental cells (S7 Fig). In addition, MDBMs expressed FMS, and exogenous M-CSF could activate FMS signaling pathways in MDBMs (Fig 6A). Therefore, a M-CSF/FMS signaling pathway is considered to stimulate osteoclast formation and osteolytic function in A549-Luc-BM1-implanted tibiae. Indeed, TAS-115 potently inhibited RANKL- and M-CSF-stimulated mouse osteoclast differentiation (Fig 5A). Interestingly, TAS-115 had more potent efficacy than cabozantinib for suppression of bone destruction and decreasing the number of osteoclasts (Figs 3 and 4C), although both compounds had almost the same inhibitory potency against VEGFRs and MET kinase (Table 1). These results suggested that FMS inhibition by TAS-115 might contribute to its potent anti-osteolytic activity in the tumor-induced bone disease model. Sunitinib, a VEGFRs inhibitor, also inhibited FMS kinase (Table 1), which was in accordance with a previous report [43]. However, single treatment of sunitinib only partially inhibited bone destruction and partially decreased the number of osteoclasts (Figs 3 and 4C), suggesting that only inhibition of FMS, or dual inhibition of VEGFRs/FMS, is not sufficient for the suppression of tumor-induced osteoclast formation and it is possible that MET signaling might complement FMS and/or VEGFR signaling in osteoclasts. To support the notion, the combined treatment of sunitinib (VEGFRs/FMS inhibition) with crizotinib (MET inhibition) exerted superior effects on bone destruction and osteoclast formation to single treatment of sunitinib (Figs 3 and 4C). These results suggest that simultaneous triple blockade of VEGFRs/MET/FMS might be required for more potent suppression of tumor-related osteoclast formation. TAS-115 could achieve simultaneous triple blockade of VEGFRs/MET/FMS by monotherapy.

It has been reported that cabozantinib significantly improves median progression-free survival and time to first SRE, compared with prednisone, suggesting that VEGFRs/MET inhibition impacts on tumor burden in castration resistant prostate cancer (CRPC) patients with bone metastasis [44]. These clinical results support the notion that both VEGFRs and MET signaling pathways are clearly involved in bone metastasis progression and that their inhibition is effective against bone metastasis. However, cabozantinib failed to significantly increase overall survival (OS) compared with prednisone in patients with metastatic CRPC in a pivotal phase III study (COMET-1). In that clinical trial, the discontinuation rate and the sequential therapies used after progression of the disease might be reasons for the failure to improve OS in the cabozantinib arm. TAS-115 significantly suppressed tumor growth without affecting body weight both in a previous study [16] and in the current study. Based on the drug potency achieved by adding FMS inhibition to VEGFRs/MET inhibition, and the good tolerability of TAS-115, we expect that TAS-115 will also exert prominent anti-tumor efficacy against bone metastasis in a clinical setting.

In conclusion, our results clearly demonstrated that TAS-115 markedly inhibited tumor growth via VEGFR-kinase blockade, and also suppressed bone destruction, possibly through VEGFRs/MET/FMS-kinase blockade, which resulted in potent efficacy of TAS-115 in the A549-Luc-BM1 bone disease model. Based on these data, TAS-115 should provide a novel therapy for patients with bone metastasis of lung or other cancer.

## Supporting Information

S1 Fig Time schedule for *in vivo* experiments. Two *in vivo* studies were separately conducted: the first study was aimed to evaluate the efficacy of drugs for tumor growth and bone lesion, the second study was to perform histopathological analyses after drug treatment. Bioluminescence imaging (IVIS) was performed to confirm the reproducibility of anti-tumor effects in the second study. Sampling for histopathological analysis was done after 2 weeks of drug treatment, because bone damage in the control groups was too severe after 4 weeks to provide histopathological analysis. Inoculation: A549-Luc-BM1 cells were implanted in mouse tibia. Allocation: A549-Luc-BM1-implanted mice were grouped into each treatment based on total photon flux. Details of the procedures are described in MATERIALS AND METHODS section.
(TIF)

S2 Fig The effect of TAS-115 on the proliferation of A549-Luc-BM1 cells. A549-Luc-BM1 cells were seeded on 96 well plates at a density of 10^3^ cells/well in RPMI1640 containing 10% FBS. The next day, TAS-115, cabozantinib, sunitinib, or crizotinib was added to the cells using increasing doses. At 72 hr post drug addition, cell viability was determined using CellTiter-Glo^TM^. The 50% growth inhibition (GI_50_) values were determined using SAS version 9.2.
(TIF)

S3 Fig Phospho-RTK array analysis of A549-Luc-BM1 cells. The cell lysate of A549-Luc-BM1 cells that were treated with or without rhHGF (100 ng/mL) was prepared and analyzed using the PathScan^®^ RTK Signaling Antibody Array Kit (#7949, CST). The open squares indicate the position of phospho-MET. The examination was conducted in duplicate.
(TIF)

S4 Fig TRAP staining of sham control tibia and disease control tibia. Instead of A549-Luc-BM1 cells, PBS was injected into mouse tibia as sham control. Disease control depicts A549-Luc-BM1 cells-implanted tibia (same picture as Fig 4C). Details of the procedures for TRAP staining are described in the MATERIALS AND METHODS section. Scale bar indicates 100 μm. B: Bone, BM: Bone marrow, T: Tumor (A549-Luc-BM1).
(TIF)

S5 Fig The inhibition of the FMS signaling pathway in human acute monocytic leukemia THP-1 cells by TAS-115. THP-1 cells were seeded in 6-well plates at a density of 2×10^6^ cells/well and TAS-115, sunitinib, crizotinib, or cabozantinib was then added at the indicated concentration. After incubation with the compounds for 120 min, THP-1 cells were stimulated with 30 ng/mL of M-CSF and lysed at 1 min post M-CSF stimulation. Specific proteins in the cell lysates were detected using immune blotting and were quantified using Multi Gauge Ver 3.2 (FUJIFILM). IC_50_ values were calculated using Xlfit 5.3.0.8 (CTC Life Science Corporation, Tokyo, Japan).
(TIF)

S6 Fig Determination of human VEGF and mouse HGF in A549-Luc-BM1-implanted tibiae. A549-Luc-BM1 implanted tibiae (n = 2/day) were removed from mice at 22, 29, and 36 days post tumor implantation, and were homogenized to prepare tissue lysates. The level of human VEGF (A) and mouse HGF (B) in the tissue lysates was determined using the human VEGF Quantikine ELISA Kit (DVE00, R&D systems) and the mouse HGF Quantikine ELISA Kit (MHG00, R&D systems), respectively. The quantified human VEGF and mouse HGF in each mouse tibia were normalized by the weight of the tibia. Normal tibiae (n = 2) were removed from mice without tumor implantation.
(TIF)

S7 Fig M-CSF expression in A549-Luc and A549-Luc-BMI cells. A549-Luc and A549-Luc-BM1 cells were seeded on 6 well plates at a density of 1.62×10^6^ and 0.97×10^6^ cells/well, respectively, in 1 mL of RPMI1640 containing 10% FBS. The next day, the level of M-CSF in the conditioned medium of both cell lines was determined using the Human M-CSF Quantikine ELISA Kit (DMC00B, R&D systems). M-CSF concentration was normalized by the number of cells. Data are expressed as means ± SD (n = 3). **, p<0.01 in the comparison of the conditioned medium of A549-Luc-BM1 cells with that of A549-Luc cells.
(TIF)

S1 Table Summary of morphometrical parameters derived from micro-CT images. (TIF)
