# Supplementary figures and images for: High Potency VEGFRs/MET/FMS Triple Blockade by TAS-115 Concomitantly Suppresses Tumor Progression and Bone Destruction in Tumor-Induced Bone Disease Model with Lung Carcinoma Cells
Source: PLoS One. 2016 Oct 13;11(10):e0164830. doi: 10.1371/journal.pone.0164830 (PMC5063576; doi:10.1371/journal.pone.0164830)

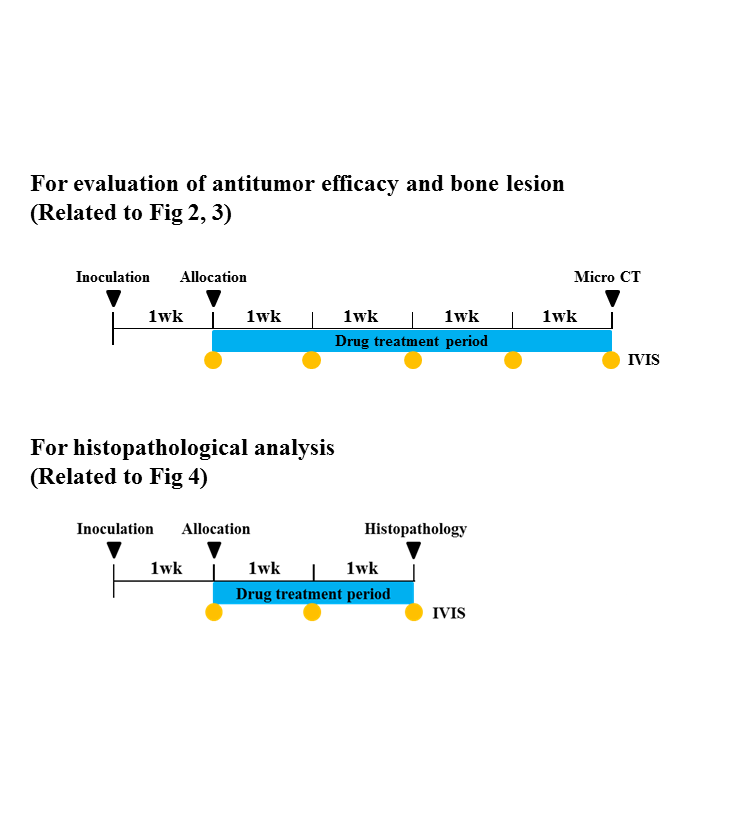

Supplement: S1 Fig — Two in vivo studies were separately conducted: the first study was aimed to evaluate the efficacy of drugs for tumor growth and bone lesion, the second study was to perform histopathological analyses after drug treatment. Bioluminescence imaging (IVIS) was performed to confirm the reproducibility of anti-tumor effects in the second study. Sampling for histopathological analysis was done after 2 weeks of drug treatment, because bone damage in the control groups was too severe after 4 weeks to provide histopathological analysis. Inoculation: A549-Luc-BM1 cells were implanted in mouse tibia. Allocation: A549-Luc-BM1-implanted mice were grouped into each treatment based on total photon flux. Details of the procedures are described in MATERIALS AND METHODS section. (TIF) [file pone.0164830.s001.tif]

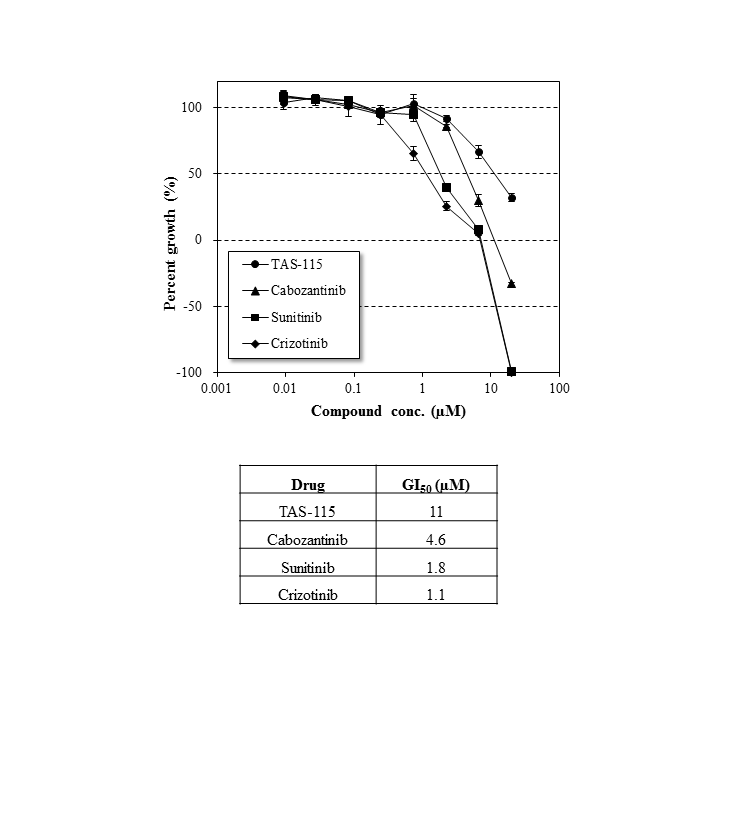

Supplement: S2 Fig — A549-Luc-BM1 cells were seeded on 96 well plates at a density of 103 cells/well in RPMI1640 containing 10% FBS. The next day, TAS-115, cabozantinib, sunitinib, or crizotinib was added to the cells using increasing doses. At 72 hr post drug addition, cell viability was determined using CellTiter-GloTM. The 50% growth inhibition (GI50) values were determined using SAS version 9.2. (TIF) [file pone.0164830.s002.tif]

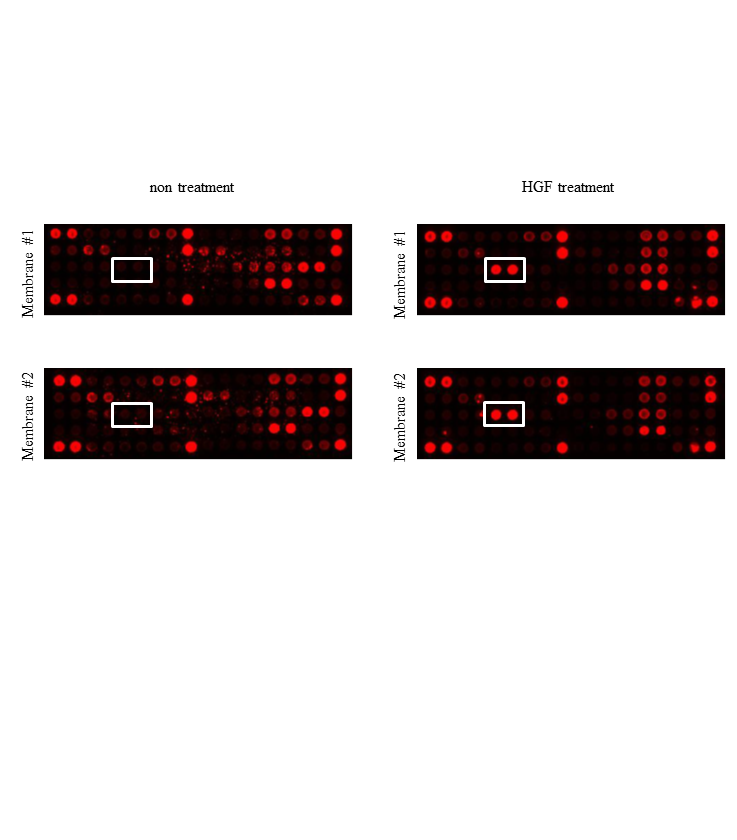

Supplement: S3 Fig — The cell lysate of A549-Luc-BM1 cells that were treated with or without rhHGF (100 ng/mL) was prepared and analyzed using the PathScan® RTK Signaling Antibody Array Kit (#7949, CST). The open squares indicate the position of phospho-MET. The examination was conducted in duplicate. (TIF) [file pone.0164830.s003.tif]

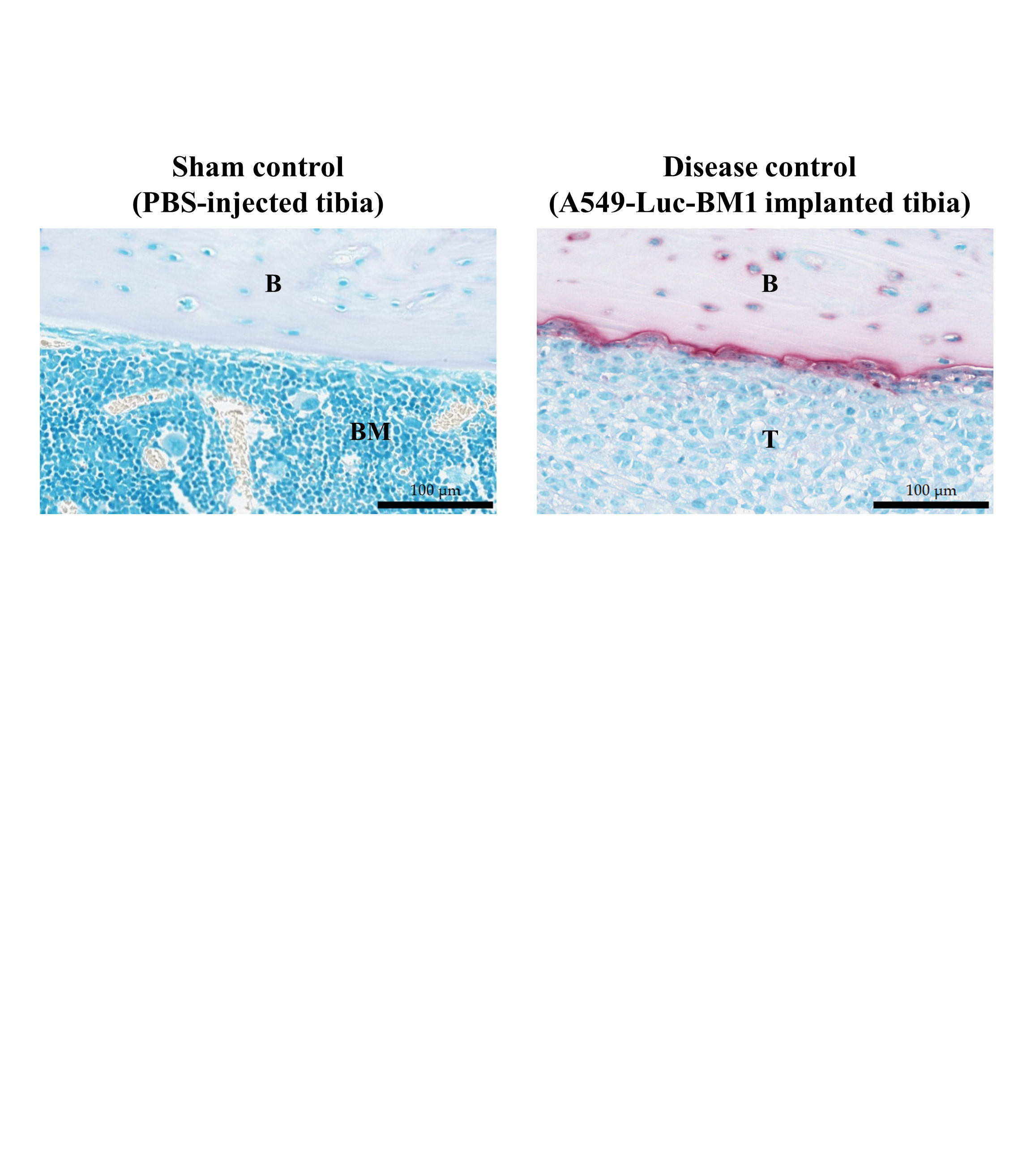

Supplement: S4 Fig — Instead of A549-Luc-BM1 cells, PBS was injected into mouse tibia as sham control. Disease control depicts A549-Luc-BM1 cells-implanted tibia (same picture as Fig 4C). Details of the procedures for TRAP staining are described in the MATERIALS AND METHODS section. Scale bar indicates 100 μm. B: Bone, BM: Bone marrow, T: Tumor (A549-Luc-BM1). (TIF) [file pone.0164830.s004.tif]

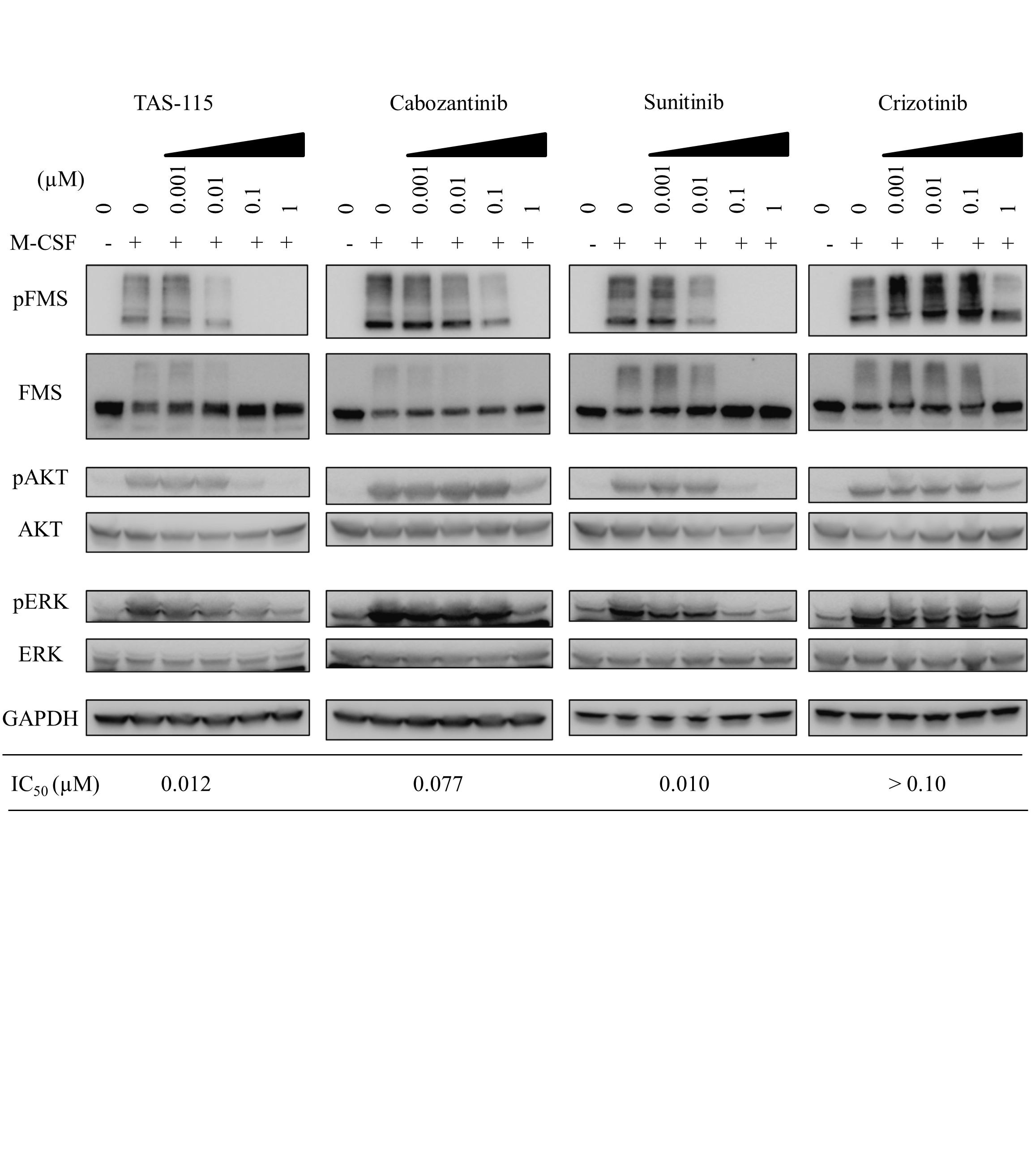

Supplement: S5 Fig — THP-1 cells were seeded in 6-well plates at a density of 2×10^6^ cells/well and TAS-115, sunitinib, crizotinib, or cabozantinib was then added at the indicated concentration. After incubation with the compounds for 120 min, THP-1 cells were stimulated with 30 ng/mL of M-CSF and lysed at 1 min post M-CSF stimulation. Specific proteins in the cell lysates were detected using immune blotting and were quantified using Multi Gauge Ver 3.2 (FUJIFILM). IC50 values were calculated using Xlfit 5.3.0.8 (CTC Life Science Corporation, Tokyo, Japan). (TIF) [file pone.0164830.s005.tif]

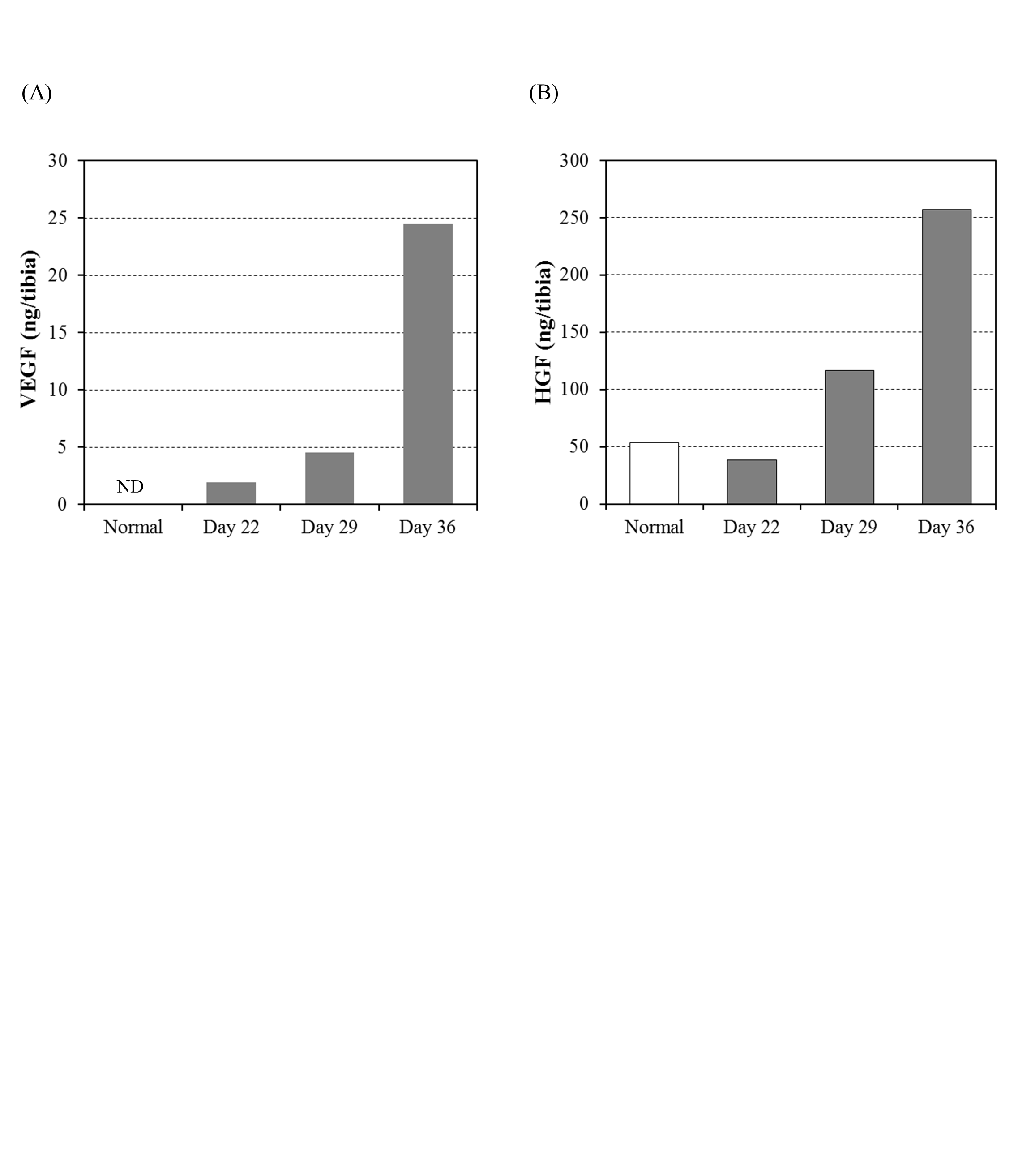

Supplement: S6 Fig — A549-Luc-BM1 implanted tibiae (n = 2/day) were removed from mice at 22, 29, and 36 days post tumor implantation, and were homogenized to prepare tissue lysates. The level of human VEGF (A) and mouse HGF (B) in the tissue lysates was determined using the human VEGF Quantikine ELISA Kit (DVE00, R&D systems) and the mouse HGF Quantikine ELISA Kit (MHG00, R&D systems), respectively. The quantified human VEGF and mouse HGF in each mouse tibia were normalized by the weight of the tibia. Normal tibiae (n = 2) were removed from mice without tumor implantation. (TIF) [file pone.0164830.s006.tif]

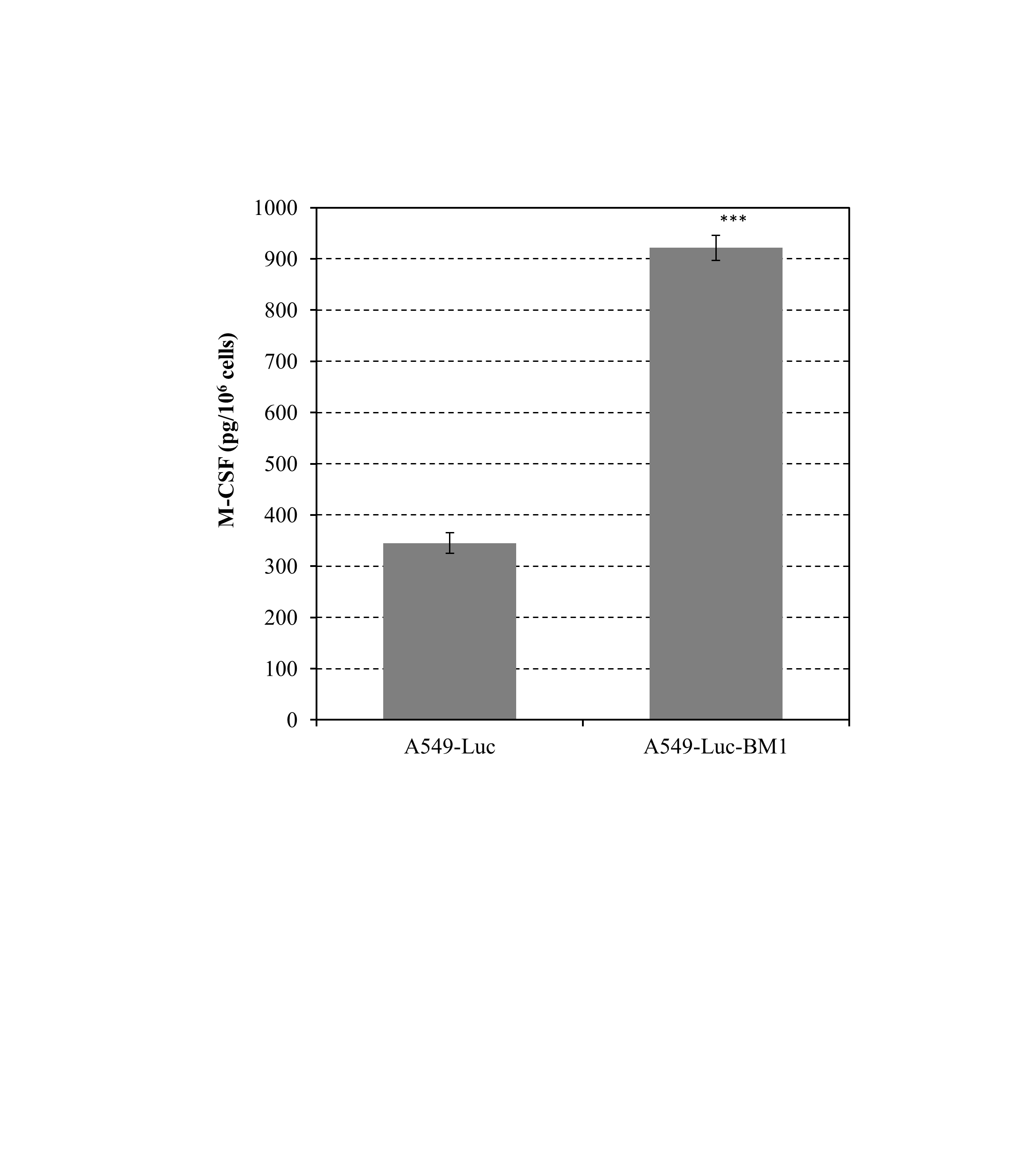

Supplement: S7 Fig — A549-Luc and A549-Luc-BM1 cells were seeded on 6 well plates at a density of 1.62×10^6^ and 0.97×10^6^ cells/well, respectively, in 1 mL of RPMI1640 containing 10% FBS. The next day, the level of M-CSF in the conditioned medium of both cell lines was determined using the Human M-CSF Quantikine ELISA Kit (DMC00B, R&D systems). M-CSF concentration was normalized by the number of cells. Data are expressed as means ± SD (n = 3). **, p<0.01 in the comparison of the conditioned medium of A549-Luc-BM1 cells with that of A549-Luc cells. (TIF) [file pone.0164830.s007.tif]

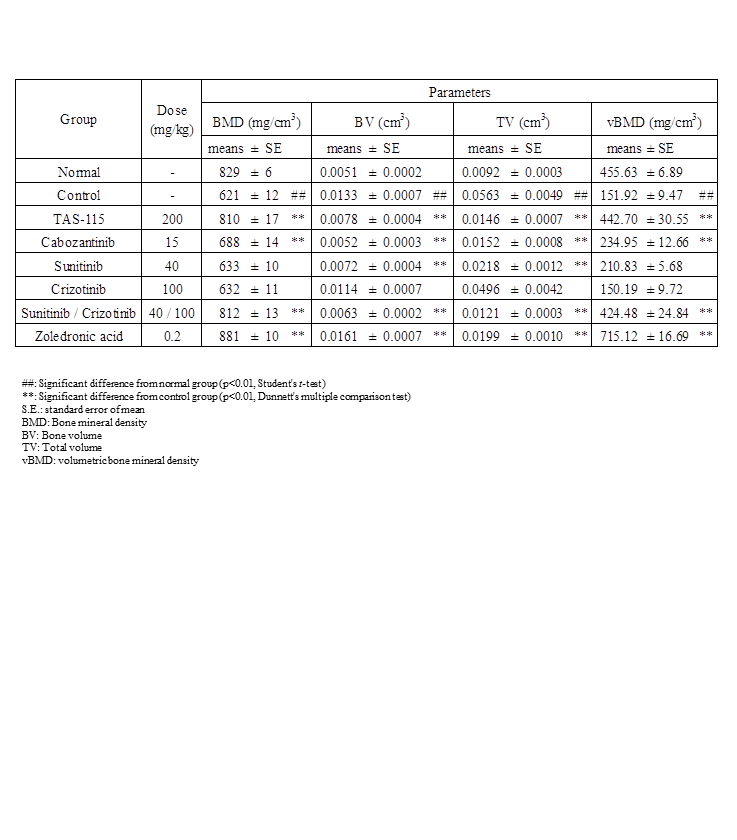

Supplement: S1 Table — (TIF) [file pone.0164830.s008.tif]
